# Dose-dependent reduction of lymphocyte count and heart rate after multiple administration of LC51-0255, a novel sphingosine-1-phosphate receptor 1 modulator, in healthy subjects

**DOI:** 10.3389/fphar.2022.930615

**Published:** 2022-08-22

**Authors:** Inyoung Hwang, Sang Won Lee, Jaeseong Oh, SeungHwan Lee, In-Jin Jang, Kyung-Sang Yu

**Affiliations:** ^1^ Department of Clinical Pharmacology and Therapeutics, Seoul National University College of Medicine and Hospital, Seoul, South Korea; ^2^ Department of Clinical Pharmacology and Therapeutics, Hanyang University Seoul Hospital, Seoul, South Korea

**Keywords:** autoimmune disease, immunosuppression, lymphocyte count, phase I, randomized controlled trial, sphingosine-1-phosphate receptor modulator

## Abstract

**Aim:** Sphingosine-1-phosphate receptor mediates the egress of lymphocytes from lymphoid organs, and its inhibition results in a decreased number of circulating lymphocytes. The aim of the current study was to investigate the safety and pharmacodynamic and pharmacokinetic characteristics of a novel sphingosine-1-phosphate receptor modulator, LC51-0255.

**Methods:** A phase 1 randomized, double-blind, placebo-controlled, multiple dosing, dose-escalation study was conducted on healthy Korean male subjects.

**Results:** After single and daily administration of LC51-0255 for 21 days, a dose-dependent decrease in lymphocyte count and heart rate was observed through 0.25–2 mg dose range of LC51-0255. The mean elimination half-life of LC51-0255 was 76–95 h. LC51-0255 was accumulated with a mean accumulation ratio of 5.17–6.64. During the study, LC51-0255 was generally well tolerated. The most common treatment-emergent adverse event was bradycardia. No clinically significant event of arrhythmia, including AV block, was observed. No clinically significant difference in blood pressure was observed between the dose groups. In other safety assessments, no clinically significant abnormalities were observed, except for bradycardia.

**Conclusion:** Daily administration of LC51-0255 in the range of 0.25–2 mg resulted in a dose-dependent reduction of lymphocyte counts and heart rate. LC51-0255 is generally safe and well tolerated in healthy volunteers.

## Introduction

Sphingosine-1-phosphate (S1P) is a bioactive sphingolipid metabolite produced by various cell types to modulate diverse physiological responses via S1P receptors (S1PR) (Alvarez et al., 2007). The mechanism of S1P receptor modulator (S1PRM) action includes the inhibition of lymphocyte egress from secondary lymphoid organs to the periphery through S1P receptor 1 (S1PR1), resulting in a decreased number of circulating lymphocytes in the peripheral blood (Matloubian et al., 2004; Cyster, 2005).

Modulation of S1PR1 has established a new approach in the treatment of autoimmune diseases, such as multiple sclerosis (MS), inflammatory bowel disease (IBD), lupus, and psoriasis (Gonzalez-Cabrera et al., 2014; Dash et al., 2018). Fingolimod is a first-in-class drug that has been approved for the treatment of relapsing-remitting MS (Park and Im, 2017). Other drugs in this category include siponimod (approved for the treatment of MS), ozanimod (approved for the treatment of MS and ulcerative colitis), and several others, which are under development (Park and Im, 2017; Al-salama, 2019; Lamb, 2020).

LC51-0255 is a newly developed potent, selective, and orally available S1PRM. LC51-0255 showed selective activity on S1PR1 among S1PR subtypes through *in vitro* Ca^2+^ mobilization assays (Ministry of Healthy and Welfare, 2014). The half maximal effective concentration (EC_50_) of LC51-0255 on S1PR1 was 21.38 nM, while >10000 and 501.19 nM on S1PR3 and S1PR5, respectively (Supplementary Table S1) (Ministry of Healthy and Welfare, 2014). In an *in vivo* pharmacology study with a rat model, a dose-dependent reduction of peripheral lymphocyte was observed after single and multiple oral administration of LC51-0255 (Ministry of Healthy and Welfare, 2014). In the rat experimental autoimmune encephalomyelitis (EAE) model, a dose-dependent reduction of EAE severity was observed (Ministry of Healthy and Welfare, 2014). In addition, recovery of intestinal tissue and dose-dependent reduction of lymphocytes in a rat model of IBD have been shown (Kim J.A et al., unpublished data, 2016). *In vitro* G protein-coupled inwardly rectifying acetylcholine-regulated K^+^ channel assay suggested that LC51-0255 might have less cardiovascular risk compared to other S1PRM (Kim et al., 2019). In the first-in-human clinical trial, a single dose of LC51-0255 of up to 2 mg was well tolerated after single administration in healthy subjects (Lee et al., 2022). The lymphocyte counts and heart rates were reduced in a reversible and dose-dependent manner (Lee et al., 2022). Based on the results of preclinical and single administration studies, LC51-0255 is expected to be a novel treatment option for patients with autoimmune and chronic inflammatory conditions.

The aim of the current study was to investigate the safety, tolerability, pharmacokinetic (PK) characteristics, and pharmacodynamic (PD) characteristics of LC51-0255 after multiple ascending oral doses for 21 days in healthy Korean male subjects.

## Materials and methods

### Study design

A phase 1, randomized, double-blind, placebo-controlled, multiple dosing, and dose-escalation study was conducted on healthy Korean male subjects between March 2018 and July 2019. The doses of LC51-0255 selected for the study were 0.25, 0.5, 1, 1.5, and 2 mg. Eligible subjects were admitted to the Seoul National University Clinical Trials Center 2 days prior to the first dosing and were randomly assigned to either LC51-0255 or a matching placebo in a ratio of 8:2. Subjects received allocated doses of LC51-0255 or a matching placebo daily for 21 days. The inpatient clinical study was completed according to the predefined schedule, and the subjects were discharged 27 days after the first dosing (day 28). Subjects visited the clinical trial center on an outpatient basis on days 30, 32, 34, 36, 38, 40, and 42 for safety monitoring and blood sampling.

All subjects provided their written informed consent prior to enrollment in the clinical study. The study protocol was reviewed and approved by the Ministry of Food and Drug Safety, Republic of Korea, and the Institutional Review Board of Seoul National University Hospital, Seoul (ClinicalTrials.gov identifier: NCT03174613). The study was conducted in accordance with the Declaration of Helsinki and Good Clinical Practice Guidelines of the International Council for Harmonization.

### Subjects

Healthy Korean male volunteers aged 19–45 years with a body mass index (BMI) of 18.0 kg/m^2^ to 27.0 kg/m^2^ at the screening visit were eligible for enrollment in the study. Subjects with evidence or a history of clinically significant hepatic, renal, neurologic, immunologic, pulmonary, endocrine, hematologic, neoplastic, cardiovascular, or psychological disease were excluded from the study.

### Determination of the LC51-0255 concentration

Plasma and urine concentrations of LC51-0255 were analyzed using a validated liquid chromatography with tandem mass spectrometry (LC-MS/MS) method (LC: Prominence UFLC, Shimadzu, Kyoto, Japan. MS: API 5000 for plasma and API 4000 for urine, SCIEX, Concord, Ontario, Canada) by International Scientific Standards, Inc. (Chuncheon, Republic of Korea). The bioanalytical methods were validated over the range of 0.3–1,000 ng/ml for plasma samples and 0.3–300 ng/ml for urine samples. The lower limits of quantification for both plasma and urine samples were 0.3 ng/ml.

### Pharmacodynamics analysis

For absolute lymphocyte count (ALC) analysis, blood samples were collected at the following time points: day −1: 0, 2, 4, 6, 8, and 12 h; day 1: 0 (predose), 2, 4, 6, 8, and 12 h (postdose); day 2–day 20 (predose); day 21: 0 (predose), 2, 4, 6, 8, 12, 24, 36, 48, 60, 72, 96, 120, 144, 168, 216, 264, 312, 360, 408, 456, and 504 h (postdose).

The maximum effect (E_max_), the area under the ALC–time curve from time zero to the last measurable point (AUEC_last_) and change of those parameters from the baseline (ΔE_max_ and ΔAUEC_last_), and the time to maximum effect (TE_max_) and maximum change of ALC from the baseline (CFB_max_) were calculated using the noncompartmental method of Phoenix WinNonlin^®^ version 8.1 (Certara, Princeton, NJ, United States).

The baseline ALC of each subject was defined using the following formula:

$$\text{Baseline ALC}=\frac{{\text{AUEC}}_{0-24}\;of\;Day-1}{24}$$

For heart rate analysis, 24-h Holter monitoring was performed the day before the first dosing (day 1), on the first dosing (day 1), and last dosing (day 21). The hourly average heart rate (HR) and the area under the hourly HR–time curve (AUEC_HR_) were calculated for each individual subject.

### Pharmacokinetics analysis

For PK evaluation, serial blood samplings were performed at the following time points: day 1: 0 (predose), 1, 2, 3, 4, 5, 6, 7, 8, 10, 12, and 24 h (postdose); day 4, 6, 8, 10, 12, 14, 16, 18, 19, and 20 (predose); and day 21: 0 (predose), 1, 2, 3, 4, 5, 6, 7, 8, 10, 12, 24, 36, 48, 72, 96, 120, 144, 168, 216, 264, 312, 360, 408, 456, and 504 h (postdose). Urine samples were collected at the following time points and intervals: day 1: 0 (predose), 0–24 h (postdose); day 21: 0 (predose), 0 to 24, 24 to 48, 48 to 72, 72 to 96, 96 to 120, 120 to 144, and 144–168 h postdose.

PK parameters were analyzed using a noncompartmental method using Phoenix WinNonlin^®^ version 8.1 (Certara, Princeton, NJ, United States). The maximum steady-state plasma concentration of LC51-0255 (C_max,ss_), the time to reach maximum plasma concentration following administration of LC51-0255 at the steady state (T_max,ss_), the area under the plasma concentration–time curve during a dosing interval at steady state (AUC_τ,ss_), the terminal half-life (t_1/2_), the accumulation ratio calculated from AUC_τ,ss_ and AUC_τ_ after single dosing (R_ac_), the peak trough fluctuation over one dosing interval at steady state, apparent clearance at steady state (CL_ss_/F), apparent volume of distribution during the terminal phase (V_z_/F), the fraction of drug excreted into urine (f_e_), and renal clearance (CL_R_) were calculated.

### Safety and tolerability assessment

Treatment-emergent adverse events (TEAEs), physical examinations, vital signs, 12-lead electrocardiogram (ECG), continuous ECG monitoring, 24-h Holter monitoring, clinical laboratory tests, pulmonary function tests, and ophthalmologic tests were performed to assess the safety and tolerability. To collect clinically significant events, the criterion for bradycardia was defined as below 40 beats per minute.

### Exploratory assessment of immune cell subset counts

Immune cell subset counts in peripheral blood were measured by fluorescence-activated cell sorting analysis using FACSVerse (BD Biosciences, Franklin Lakes, NJ, United States) by Meditree Co., Ltd. (Seoul, Republic of Korea). The details of antibodies used are presented in Supplementary Table S2. Blood samples for analysis were collected at day 1 (predose), day 8 (4 h postdose), and day 21 (4 h postdose).

### Statistical analysis

Descriptive statistics were used to summarize demographic, PK, PD, and safety data. All statistical analyses were performed using SAS^®^ version 9.4 (SAS Institute, Inc., Cary, NC, United States). The Kruskal–Wallis test was performed to compare the demographic characteristics between the treatment groups. ANOVA using the general linear model and Dunnett’s method for multiple comparison tests was performed to compare PD parameters between the treatment groups. Pearson’s correlation coefficient was used to assess the correlation between PK and PD parameters.

## Results

### Study population

A total of 50 subjects were enrolled, and 46 subjects completed the study. Three subjects withdrew their consent after receiving LC51-0255 1 mg, 1.5 mg, and 2 mg, respectively, and discontinued from the study citing personal reasons. One subject was discontinued from the study by the investigator due to noncompliance with the study instructions after receiving LC51-0255 2 mg (Supplementary Figure S1).

Mean ± standard deviation (SD) values of age, weight, height, and BMI were 30.5 ± 5.12 years, 70.8 ± 7.86 kg, 1.75 ± 0.05 m, and 23.2 ± 2.28 kg/m^2^, respectively (Table 1). No statistically significant difference in demographic characteristics was observed among the treatment groups (*p* > 0.05).

**TABLE 1:** Summary of demographic characteristics.

Demographic characteristics	0.25 mg	0.5 mg	1 mg	1.5 mg	2 mg	Placebo	Total	*p*-value
Age (year)	30.1 ± 5.33	28.0 ± 5.50	29.0 ± 4.78	33.0 ± 6.32	31.5 + 3.51	31.0 + 4.90	30.5 ± 5.12	0.4336
Weight (kg)	74.1 ± 6.06	73.0 ± 8.57	71.1 ± 5.69	68.1 ± 9.25	72.6 + 9.63	66.8 + 6.76	70.8 ± 7.86	0.3391
Height (m)	1.77 ± 0.07	1.76 ± 0.04	1.73 ± 0.07	1.72 ± 0.04	1.75 + 0.04	1.75 ± 0.04	1.75 ± 0.05	0.4879
BMI (kg/m^2^)	23.8 ± 1.75	23.6 ± 2.37	23.8 ± 2.17	23.0 ± 2.95	23.5 + 2.5	21.9 + 1.84	23.2 ± 2.28	0.5755

**Notes:** All data are presented as mean ± standard deviation. *p*-value was calculated using the Kruskal–Wallis test to compare the demographic characteristics between the treatment groups. **Abbreviation:** BMI, body mass index.

### Pharmacodynamics

#### Absolute lymphocyte count

**FIGURE 1:**
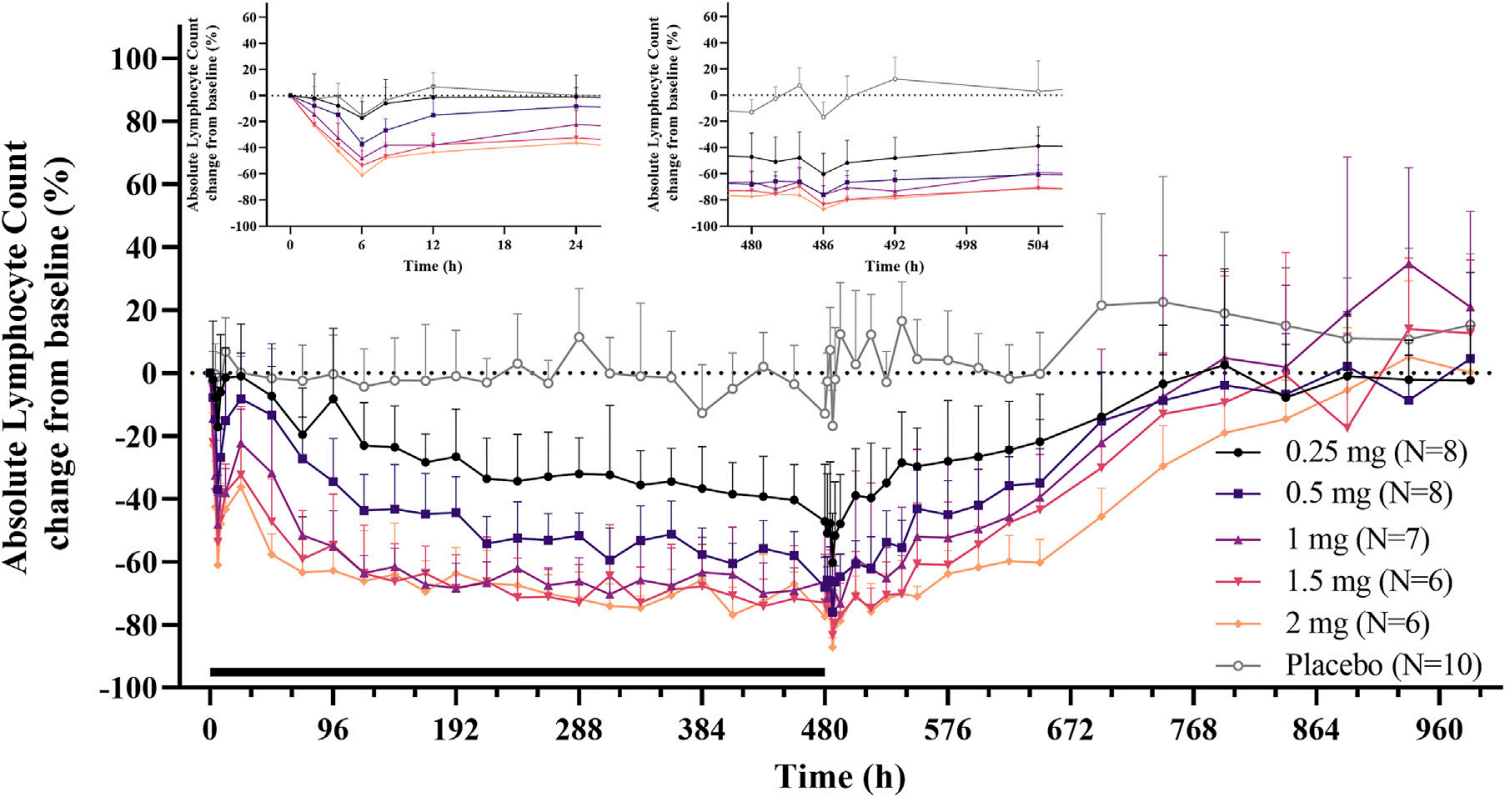
Mean absolute lymphocyte count (change from the baseline) profiles after daily oral administration of 0.25, 0.5, 1, 1.5, and 2 mg LC51-0255 for 21 days. Notes: Error bars denote the standard deviations. The black bar represents the duration of treatment. The inset graphs show the enlarged 24-h profiles after the first and last dose.

**FIGURE 2:**
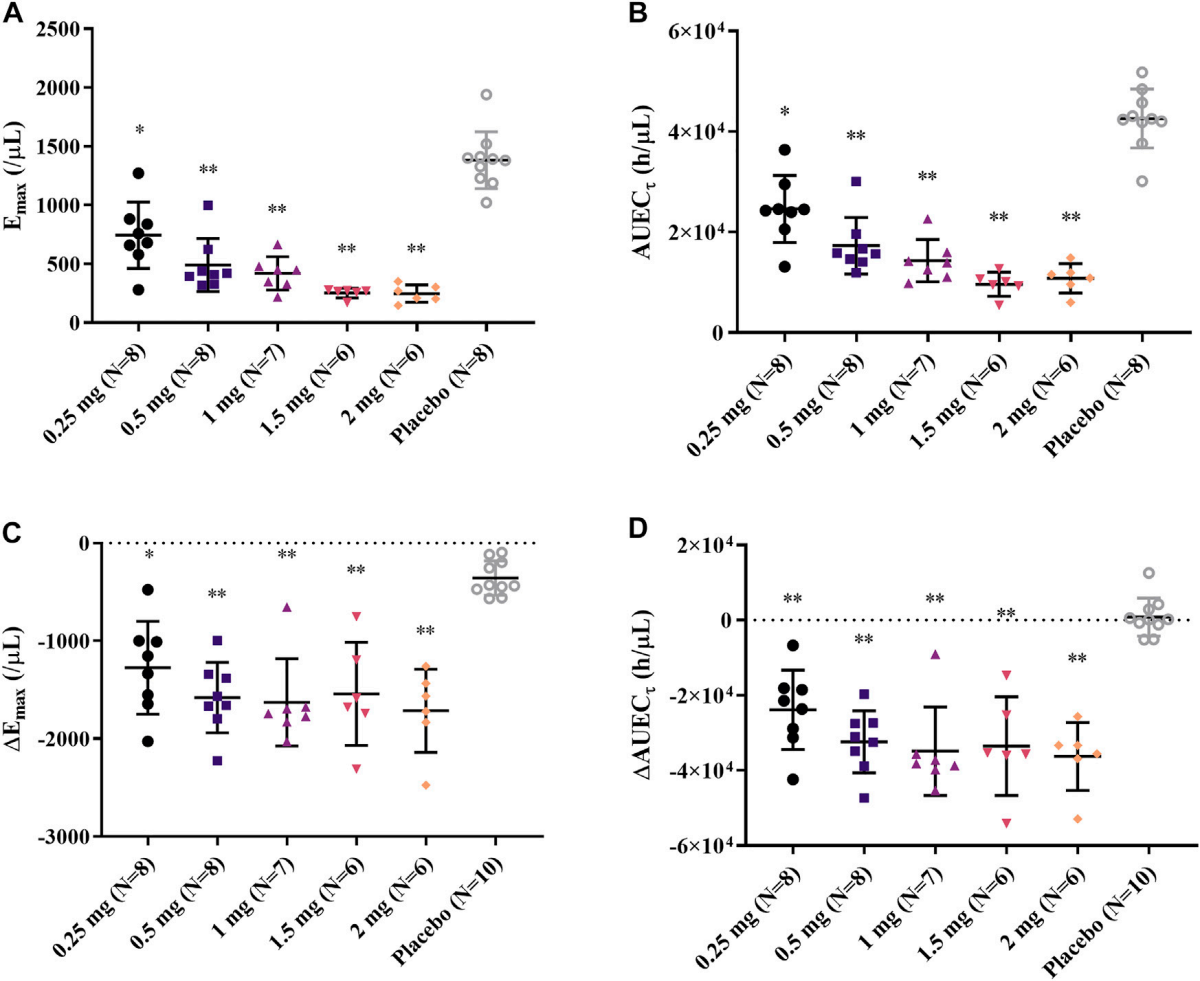
Individual **(A)** E_max_, **(B)** AUEC_τ_, **(C)** ΔE_max_, and **(D)** ΔAUEC_τ_ after daily oral administration of 0.25, 0.5, 1, 1.5, and 2 mg LC51-0255 for 21 days. Notes: Middle line: mean value; upper and lower lines: standard deviations; ∗*p* < 0.05, versus placebo and ∗∗*p* < 0.0001, versus placebo.

After single and daily administration of LC51-0255 for 21 days, a dose-dependent decrease of ALC was observed through 0.25–2 mg dose range of LC51-0255 (Figure 1). The mean ± SD values for the maximum change in ALC from the baseline (CFB_max_) were −61.84 ± 14.70%, −76.70 ± 5.60%, −82.12 ± 3.68%, −85.30 ± 6.41%, and −87.98 ± 3.50% in the LC51-0255 0.25, 0.5, 1, 1.5, and 2 mg dose groups, respectively, which were significantly lower than that in the placebo group (−31.57 ± 11.17%) (Table 2). Other PD parameters (E_max_ and AUEC_τ_) and baseline-corrected parameters were also significantly lower in all the dose groups than in the placebo group (Figure 2). All dose levels reached their maximum PD effect at 6 h postdose, and ALC recovered to the baseline within 14 days of drug discontinuation (Table 2).

**TABLE 2:** Pharmacodynamic parameters of LC51-0255 after daily oral administration of 0.25, 0.5, 1, 1.5, 2 mg of LC51-0255 or placebo for 21 days.

PD parameter	Period	0.25 mg (N = 7)	0.5 mg (N = 8)	1 mg (N = 7)	1.5 mg (N = 6)	2 mg (N = 6)	Placebo (N = 10)
TE_max_ (h)	Baseline	1.00 (0.00–23.92)	1.00 (0.00–8.00)	4.00 (0.00–8.00)	2.00 (0.00–2.00)	2.00 (0.00–23.58)	6.00 (0.00–23.83)
	Day 1	6.01 (2.00–8.02)	6.00 (6.00–23.85)	6.00 (6.00–12.00)	6.00 (6.00–6.00)	6.00 (6.00–6.05)	6.00 (2.00–12.02)
	Day 21	6.00 (2.00–12.00)	6.00 (0.00–8.00)	6.00 (0.00–8.00)	6.00 (2.00–24.02)	6.00 (0.00–6.00)	6.00 (0.00–8.00)
E_max_ (/μL)	Baseline	1,656 ± 324	1,655 ± 287	1,506 ± 262	1,447 ± 381	1,619 ± 415	1,412 ± 195
	Day 1	1,556 ± 258	1,305 ± 353	1,037 ± 290	791 ± 135	764 ± 226	1,440 ± 275
	Day 21	744 ± 283	491 ± 225	420 ± 142	253 ± 41	247 ± 74	1,382 ± 241
AUEC_τ_ (h/μL)	Baseline	48,419 ± 10,211	49,678 ± 12,859	49,147 ± 8,844	43,062 ± 12,080	47,060 ± 11,272	41,718 ± 4,632
	Day 1	45,941 ± 8,852	41,566 ± 10,409	33,736 ± 6,260	26,531 ± 5,607	28,450 ± 9,624	39,356 ± 7,704
	Day 21	24,612 ± 6,663	17,304 ± 5,609	14,311 ± 4,190	9,622 ± 2,388	10,815 ± 2,923	42,563 ± 5,859
ΔE_max_ (/μL)	Day 1	-461 ± 260	-765 ± 212	-1,011 ± 194	-1,003 ± 432	-1,197 ± 322	-298 ± 179
	Day 21	-1,274 ± 476	-1,579 ± 361	-1,628 ± 446	-1,541 ± 528	-1714 ± 424	-357 ± 176
ΔAUEC_τ_ (h/μL)	Day 1	-2,133 ± 3,859	-7,750 ± 3,343	-15067 ± 3,232	-16197 ± 8,252	-18351 ± 3,582	-7 ± 2,271
	Day 21	-23846 ± 10,577	-32381 ± 8,284	-34836 ± 11,766	-33479 ± 13,130	-36250 ± 9,042	843 ± 5,068
CFB_max_ (%)		-61.84 ± 14.70	-76.70 ± 5.60	-82.12 ± 3.68	-85.30 ± 6.41	-87.98 ± 3.50	-31.57 ± 11.17

**Notes:** All data are presented as mean ± standard deviation, except for TE_max_, which is presented as median (minimum–maximum). **Abbreviations:** PD, pharmacodynamic; TE_max_, time to maximum effect; E_max_, maximum effect; AUEC_τ_, area under the ALC–time curve during a dosing interval; ΔE_max_, change of E_max_ from the baseline; ΔAUEC_τ_ change of AUEC_τ_ from the baseline; and CFB_max_, maximum change of ALC from the baseline.

### Heart rate

**FIGURE 3:**
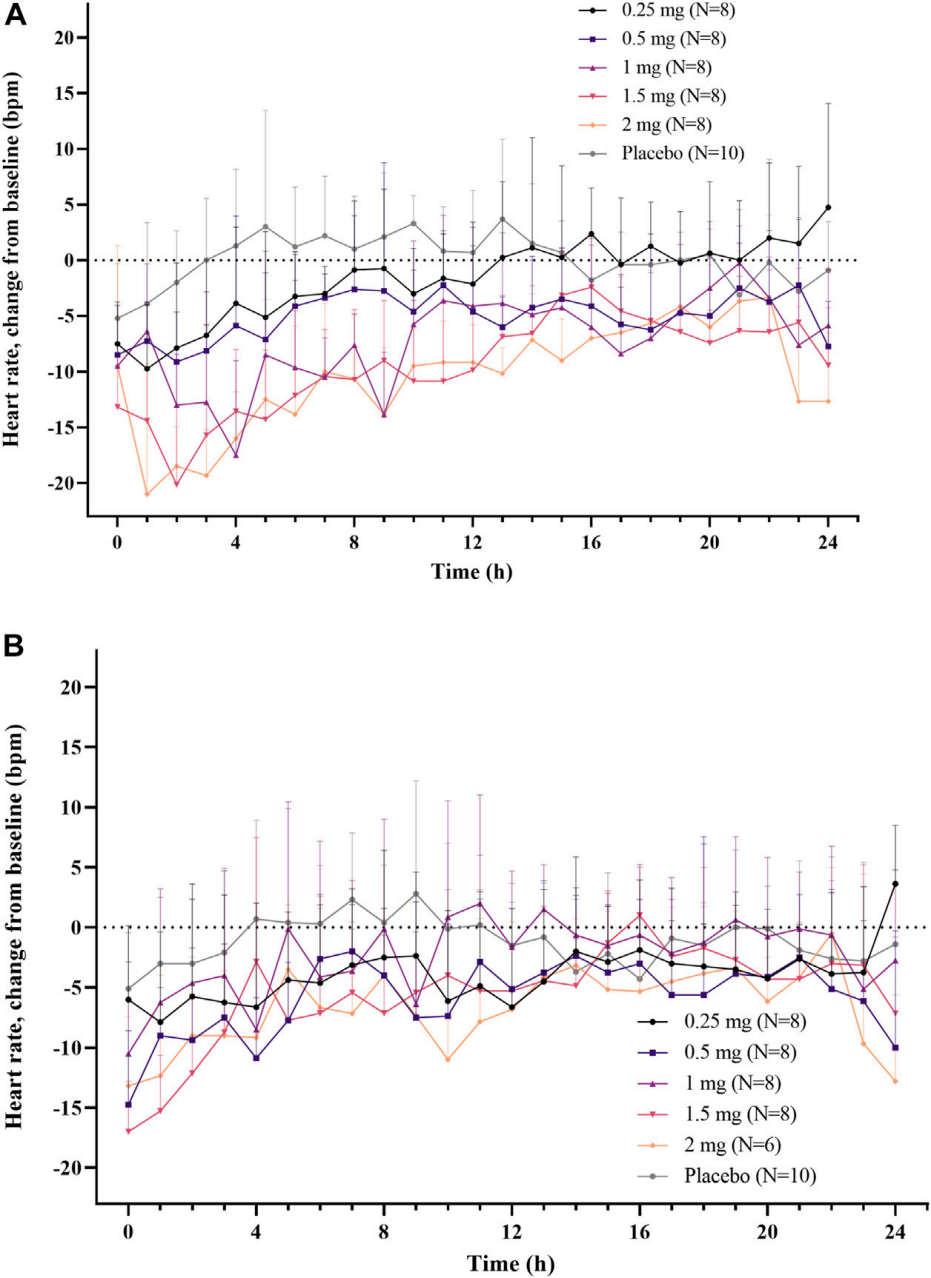
Mean hourly average heart rate (change from the baseline) after **(A)** single oral administration and **(B)** daily oral administration of 0.25, 0.5, 1, 1.5, and 2 mg of LC51-0255 for 21 days. Note: Error bars denote the standard deviations.

**FIGURE 4:**
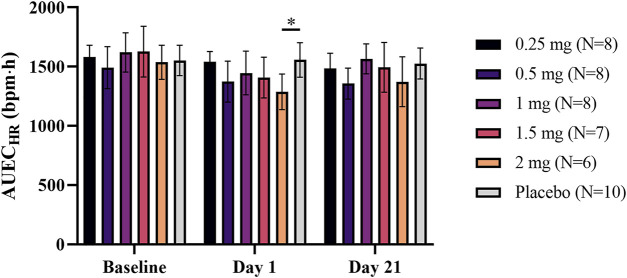
Mean area under effect curve of hourly average heart rate at the baseline, after single and daily oral administration of 0.25, 0.5, 1, 1.5, and 2 mg of LC51-0255 for 21 days. Notes: Error bars denote the standard deviations; ∗*p* < 0.05, LC51-0255 2 mg versus placebo at day 1.

A dose-dependent reduction in HR was observed after a single administration of LC51-0255 (0.25–2 mg) (Figure 3A). A statistically significant decrease in AUEC_HR_ compared to the placebo group was observed in the 2 mg dose group (Figure 4). After daily administration of LC51-0255 for 21 days, the reduction in HR was insignificant (Figure 3B). No dose group showed a statistically significant decrease in AUEC_HR_ compared to the placebo group (Figure 4).

### Pharmacokinetics

**FIGURE 5:**
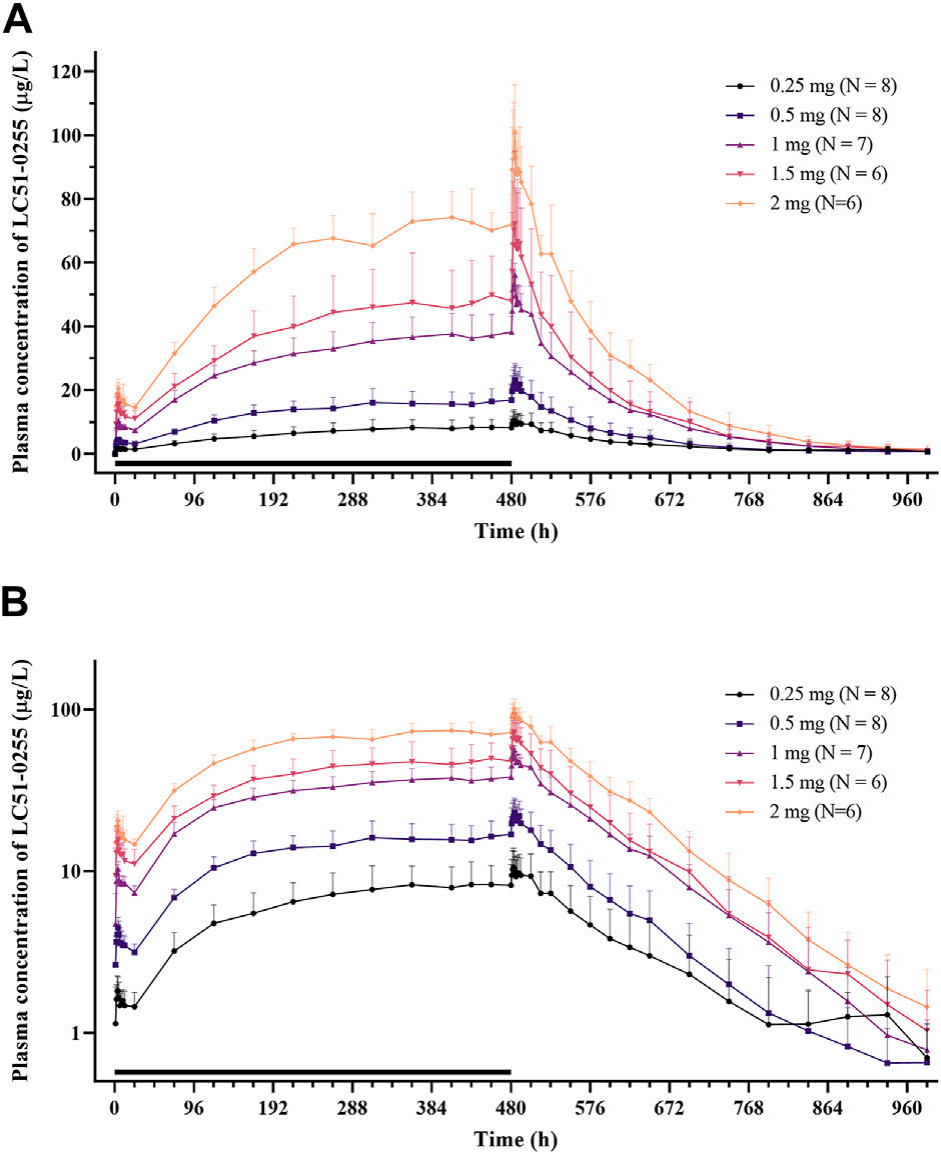
Mean plasma concentration–time profiles of LC51-0255 in **(A)** linear scale and **(B)** log linear scale after daily oral administration of 0.25, 0.5, 1, 1.5, and 2 mg of LC51-0255 for 21 days. Notes: Error bars denote the standard deviations. The black bar represents the duration of treatment.

After daily administration of LC51-0255 for 21 days, the peak plasma concentration at the steady state was observed at 4–4.5 h postdose, and the mean elimination half-life was 76–95 h (Figure 5; Table 3). LC51-0255 accumulated considerably with a mean accumulation ratio of 5.17–6.64 across the dose groups. Systemic exposure to LC51-0255 was proportional to the dose. The fraction excreted into urine ranged from 0.01 to 0.3% across the dose levels, indicating that renal excretion was not the major route of elimination.

**TABLE 3:** Pharmacokinetic parameters of LC51-0255 after daily oral administration of 0.25, 0.5, 1, 1.5, or 2 mg of LC51-0255 for 21 days.

PK parameter	0.25 mg (N = 7)	0.5 mg (N = 8)	1 mg (N = 7)	1.5 mg (N = 6)	2 mg (N = 6)
T_max,ss_ (h)	4.00 (1.00–24.0)	4.51 (4.00–10.0)	4.00 (2.00–8.00)	4.00 (3.05–12.0)	4.00 (4.00–10.0)
C_max,ss_ (μg/L)	11.1 ± 3.28	23.6 ± 5.29	57.3 ± 3.82	73.5 ± 22.1	103 ± 12.3
AUC_τ,ss_ (h* μg/L)	229 ± 67.2	478 ± 115	1,120 ± 125	1,460 ± 412	2060 ± 260
t_1/2_ (h)	95 ± 43	81 ± 22	78 ± 10	76 ± 12	79 ± 11
R_ac_	6.64 ± 2.13	6.06 ± 1.77	5.88 ± 0.617	5.17 ± 0.698	5.53 ± 0.84
PTF (%)	20.5 ± 17.1	29.4 ± 5.94	30.3 ± 18.3	33.8 ± 6.61	28.5 ± 5.04
CL_ss_/F (L/h)	1.18 ± 0.334	1.1 ± 0.254	0.906 ± 0.104	1.09 ± 0.265	0.985 ± 0.13
V_z_/F (L)	131 ± 30.6	126 ± 39.5	108 ± 15.3	112 ± 26.2	179 ± 123

**Notes:** All data are presented as mean ± standard deviation, except for T_max,ss_, which is presented as median (minimum–maximum). The half-life (t_1/2_) was calculated by computing the best fit line using Phoenix WinNonlin^®^. **Abbreviations:** T_max,ss_, the time to reach maximum plasma concentration at steady state; C_max,ss_, maximum steady-state plasma concentration of LC51-0255; AUC_τ, ss_, area under the plasma concentration–time curve during a dosing interval at steady state; t_1/2_, terminal half-life; R_ac_, accumulation ratio calculated from AUC_τ_,_ss_ and AUC_τ_ after single dosing; PTF, peak trough fluctuation over one dosing interval at steady state; CL_ss_/F, apparent clearance at steady state; and V_z_/F, apparent volume of distribution during the terminal phase.

### Relationship between pharmacokinetics and pharmacodynamics

Baseline-corrected PD parameters (ΔE_max_ and ΔAUEC_τ_) were significantly correlated with selected PK parameters (C_max_ and AUC_τ_) after the first administration of LC51-0255 (Supplementary Figure S2). After daily administration for 21 days, the correlation was weaker compared to that after the first day (Supplementary Figure S3).

### Safety and tolerability

Overall, 89 TEAEs were reported in 34 subjects (68.0%). The most common TEAE was bradycardia, which occurred 14 times in a total of 8 subjects (16%) (Supplementary Table S3). No clinically significant event of arrhythmia, including AV block, was observed. No clinically significant difference in blood pressure was observed between the dose groups (Supplementary Figure S4).

One serious adverse event (SAE) was reported in the LC51-0255 1 mg dose group. One subject was diagnosed with colonic diverticulitis on day 21, which required intravenous antibiotics for treatment. The SAE resolved without sequelae.

In the clinical laboratory test, pulmonary function test, and ophthalmologic test, no clinically significant abnormalities or changes were observed. In ECGs, Holter monitoring, and vital signs, no clinically significant abnormalities were observed, except for bradycardia.

### Explorative analysis of immune cell subset counts

A dose-dependent decrease in immune cell subset counts (CD3^+^, CD4^+^, CD8^+^, CD14^+^, and CD19^+^) was observed across all the dose groups. The reduction in CD4^+^ (helper T cells) counts was the most significant, while the reduction in CD14^+^ (myeloid cells) counts was relatively mild. The CD4+/CD8+ ratio was also reduced in a dose-dependent manner (Supplementary Figure S5).

## Discussion

This study investigated the effect of LC51-0255 on ALC and HR, along with PK, safety, and tolerability profiles of LC51-0255 after daily oral administration for 21 days in a dose range of 0.25–2 mg.

As expected from other S1P agonists such as fingolimod, multiple administrations of LC51-0255 resulted in a lower nadir ALC (E_max_) than that after a single administration (Kovarik et al., 2004). The E_max_ value ranged from 744/μL to 247/μL after daily administration of LC51-0255 for 21 days, with a tendency to decrease at higher doses (i.e., greater absolute ΔE_max_ values). The maximum change from the baseline exceeded 80% at LC51-0255 dose higher than 1 mg and was comparable to the standard dose of fingolimod (Francis et al., 2014). Three subjects (one in the 1.5 mg dose group and two in the 2 mg dose group) were reported to have severe lymphopenia (ALC <200 cells/μL) after LC51-0255 treatment. As severe lymphopenia after S1PRM therapy was manageable and was not related to SAEs in the previous clinical trials, the risk of opportunistic infection after administration of LC51-0255 was regarded as minimal (Chun et al., 2021).

In the case of HR, the first dose-related reduction of HR was observed, which is a well-known class effect (Dimarco et al., 2010). S1PR-dependent activation of G-protein-coupled inwardly rectifying potassium channels of atrial myocytes leads to a negative chronotropic effect and delayed atrioventricular conduction (Camm et al., 2014). LC51-0255 was thought to be less prone to the first-dose effect due to a long half-life, an effect called “built-in up-titration,” which was observed in S1PRMs with long half-lives, such as fingolimod (Juif et al., 2016). First, dose-related bradycardia observed in the study was mostly asymptomatic and was not clinically significant. Therefore, the need for an up-titration regimen was considered minimal.

The correlation between PK parameters (C_max_ and AUC_τ_) and the baseline-corrected PD parameters (ΔE_max_ and ΔAUEC_τ_) was observed after the first administration but was weaker after repeated administration. It was considered to be due to the range of exposure which was relatively wider while the effect was rapidly saturated after repeated administration of LC51-0255.

During the study, one case of diverticulitis was reported as an SAE. Multiple genetic and environmental factors might contribute to the etiology of diverticulitis (Reichert and Lammert, 2015). As reduction of lymphocytes might have affected the occurrence and progression of the condition, the causal relationship between diverticulitis and LC51-0255 was not ruled out. Besides bradycardia, which was predicted from the clinical studies of fingolimod, no case of clinically significant arrhythmia was reported during the study.

In contrast to fingolimod, a nonselective S1P modulator, LC51-0255 did not show agonistic activity for S1PR3 in preclinical studies. Therefore, the risk of adverse events related to vasoconstriction (hypertension, headache, and stroke) which may be associated with S1PR3 was predicted to be low (Willis and Cohen, 2013; Camm et al., 2014; Stepanovska et al., 2020). Although the duration of the study was relatively short and the number of subjects was considered small to fully evaluate the frequency of such vasoconstrictive events, no event of stroke or clinically significant change in blood pressure was observed during the study. In addition, the ratio of a subject with headache was comparable between the placebo group (2/10, 20%) and the LC51-0255 treated group (5/40, 12.5%). Further evaluation of S1PR3-related adverse events is required in future studies.

Exploratory analysis of the immune cell subsets suggested that LC51-0255 affected CD4^+^ T cells the most, as the greatest decrease was observed. The CD4+/CD8+ cell ratio was also significantly reduced after the daily administration of LC51-0255. The extent of reduction increased with the dose of LC51-0255 and the length of dosing. Similar changes in lymphocyte subsets were observed in fingolimod-treated MS patients (Rudnicka et al., 2015). Cases of viral infections in patients treated with fingolimod might be correlated with impaired cellular immunity to suppress viral infection caused by such imbalances in lymphocyte subsets (Uccelli et al., 2011; Gross et al., 2012; Ratchford et al., 2012).

Restriction of the study subject to Korean ethnicity was one of the limitations of the study. As fingolimod did not show a marked difference in PK, lymphocyte trafficking, and HR responses between ethnic groups, the conclusion drawn from the study was considered sufficiently robust (Kovarik et al., 2007). Further studies should consider including subjects from various ethnic groups to generalize the result across ethnic groups.

In conclusion, daily administration of LC51-0255 in the range of 0.25–2 mg to healthy volunteers resulted in a dose-dependent reduction in lymphocyte counts. The decrease in HR after the first administration was also dose dependent; however, it was insignificant after repeated dosing. PK features, including a long half-life, favored the once-daily dosing regimen. LC51-0255 was generally safe and well tolerated during the study. The result of the study warrants further evaluation of the efficacy and safety of LC51-0255 for patients with autoimmune and chronic inflammatory diseases.

## Data Availability

The raw data supporting the conclusion of this article will be made available by the authors, without undue reservation.
