# Supplementary material for: Dose-dependent reduction of lymphocyte count and heart rate after multiple administration of LC51-0255, a novel sphingosine-1-phosphate receptor 1 modulator, in healthy subjects
Source: Front Pharmacol. 2022 Aug 22;13:930615. doi: 10.3389/fphar.2022.930615 (PMC9442045; doi:10.3389/fphar.2022.930615)
Supplement: Supplementary file 1 [file DataSheet1.docx]

Supplementary Material

#
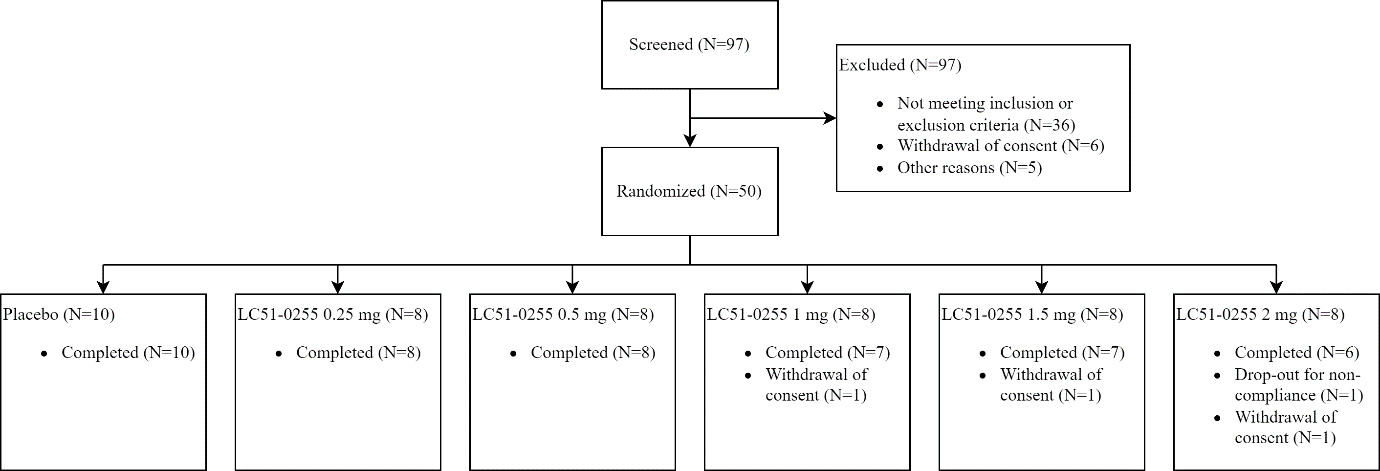


**Supplementary Figure 1.** Flow diagram of study population.


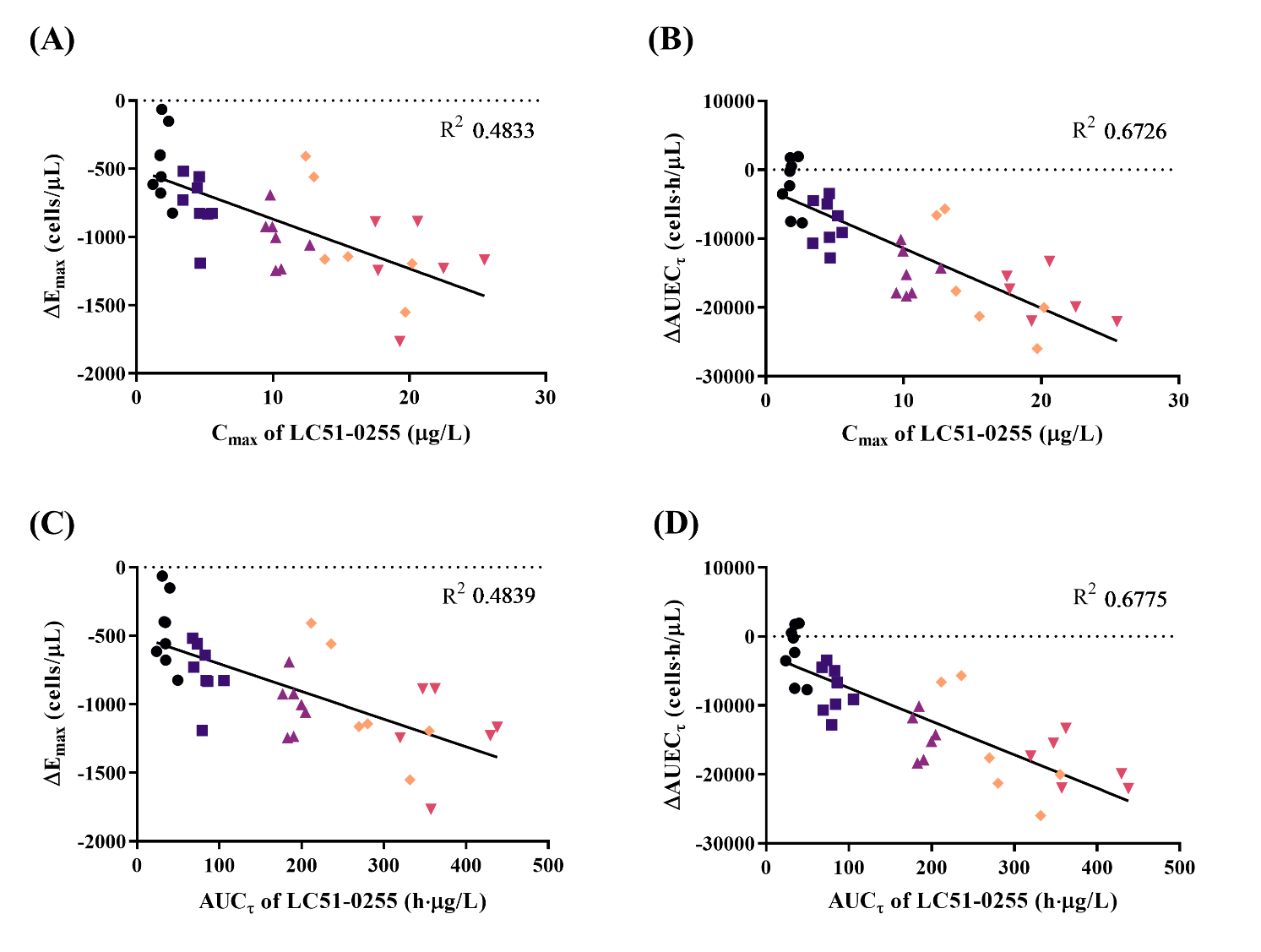


**Supplementary Figure 2.** Linear regression of relationship between selected PK parameters (C_max_, AUC_τ_) and PD parameters (ΔE_max_, ΔAUEC_τ_) after single oral administration of 0.25 mg, 0.5 mg, 1 mg, 1.5 mg, and 2 mg LC51-0255. (A) C_max_ versus ΔE_max_, (B) C_max_ versus ΔAUEC_τ_, (C) AUC_τ_ versus ΔE_max_ and (D) AUC_τ_ versus ΔAUEC_τ_.


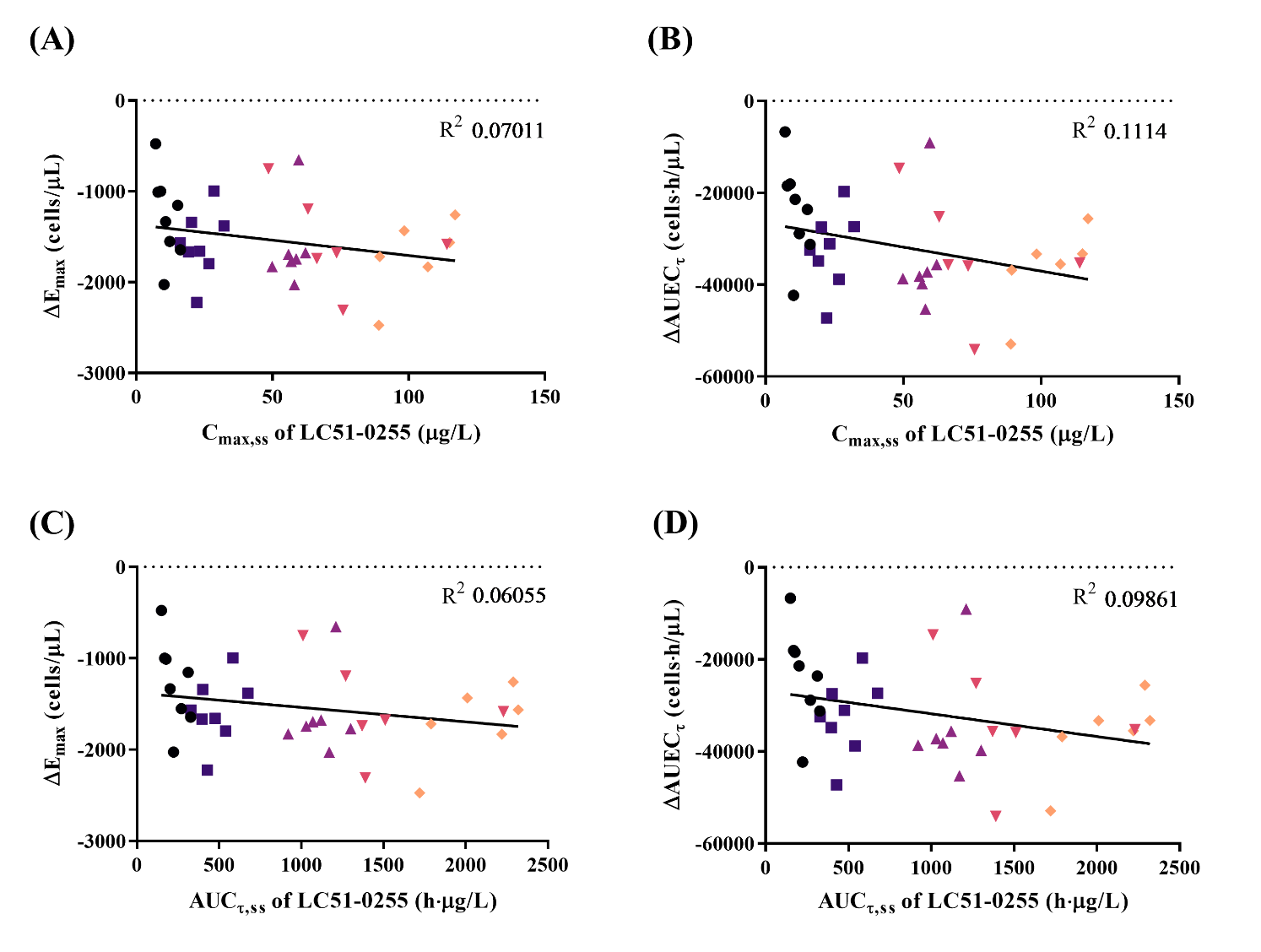


**Supplementary Figure 3.** Linear regression of relationship between selected PK parameters (C_max,ss_, AUC_τ,ss_) and PD parameters (ΔE_max_, ΔAUEC_τ_) after daily oral administration of 0.25 mg, 0.5 mg, 1 mg, 1.5 mg, and 2 mg LC51-0255 for 21 days. (A) C_max_ versus ΔE_max_, (B) C_max_ versus ΔAUEC_τ_, (C) AUC_τ_ versus ΔE_max_ and (D) AUC_τ_ versus ΔAUEC_τ_.


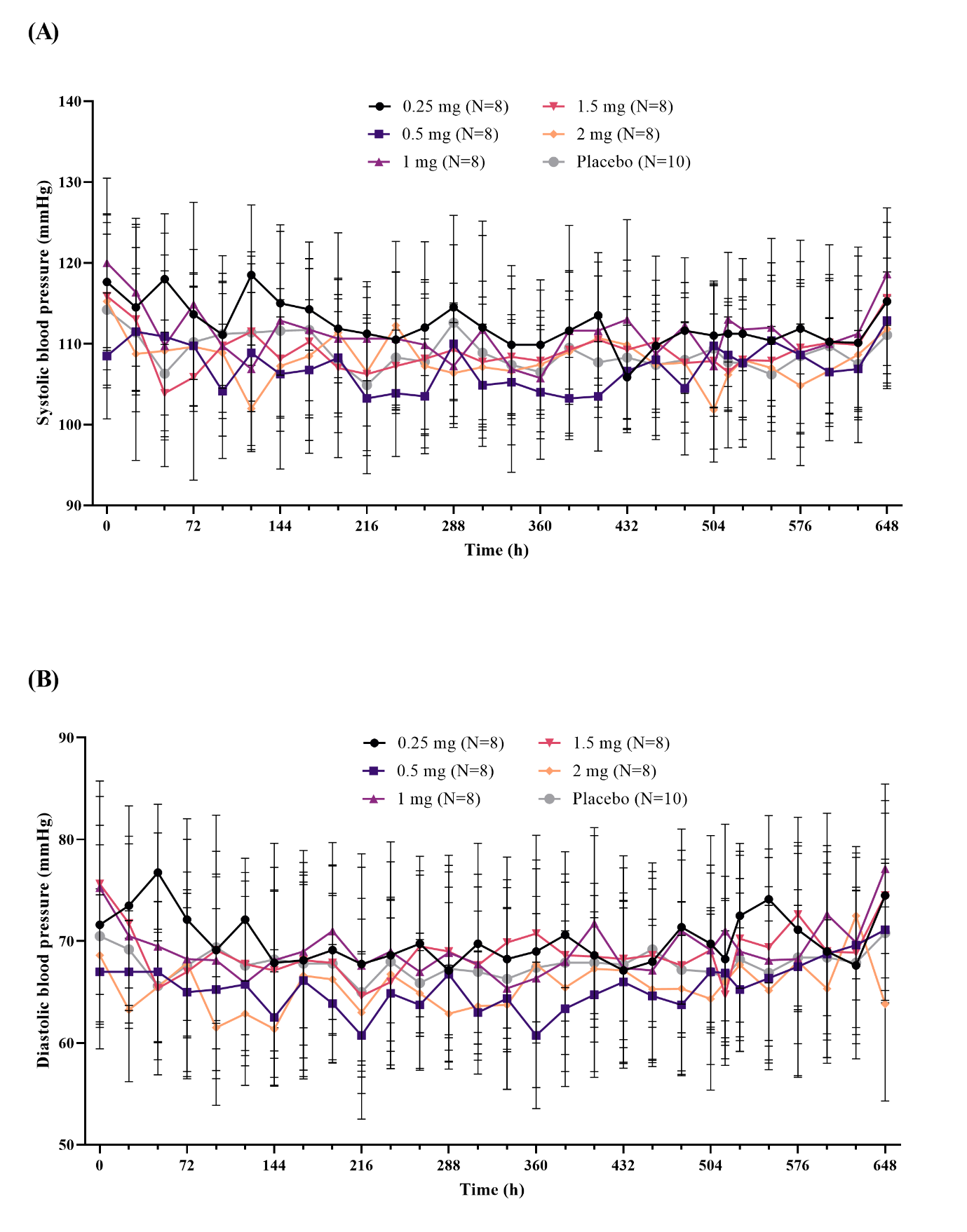


**Supplementary Figure 4.** Mean (A) systolic blood pressure and (B) diastolic blood pressure after daily oral administration of 0.25 mg, 0.5 mg, 1 mg, 1.5 mg, and 2 mg LC51-0255 for 21 days. Error bars denote the standard deviations.


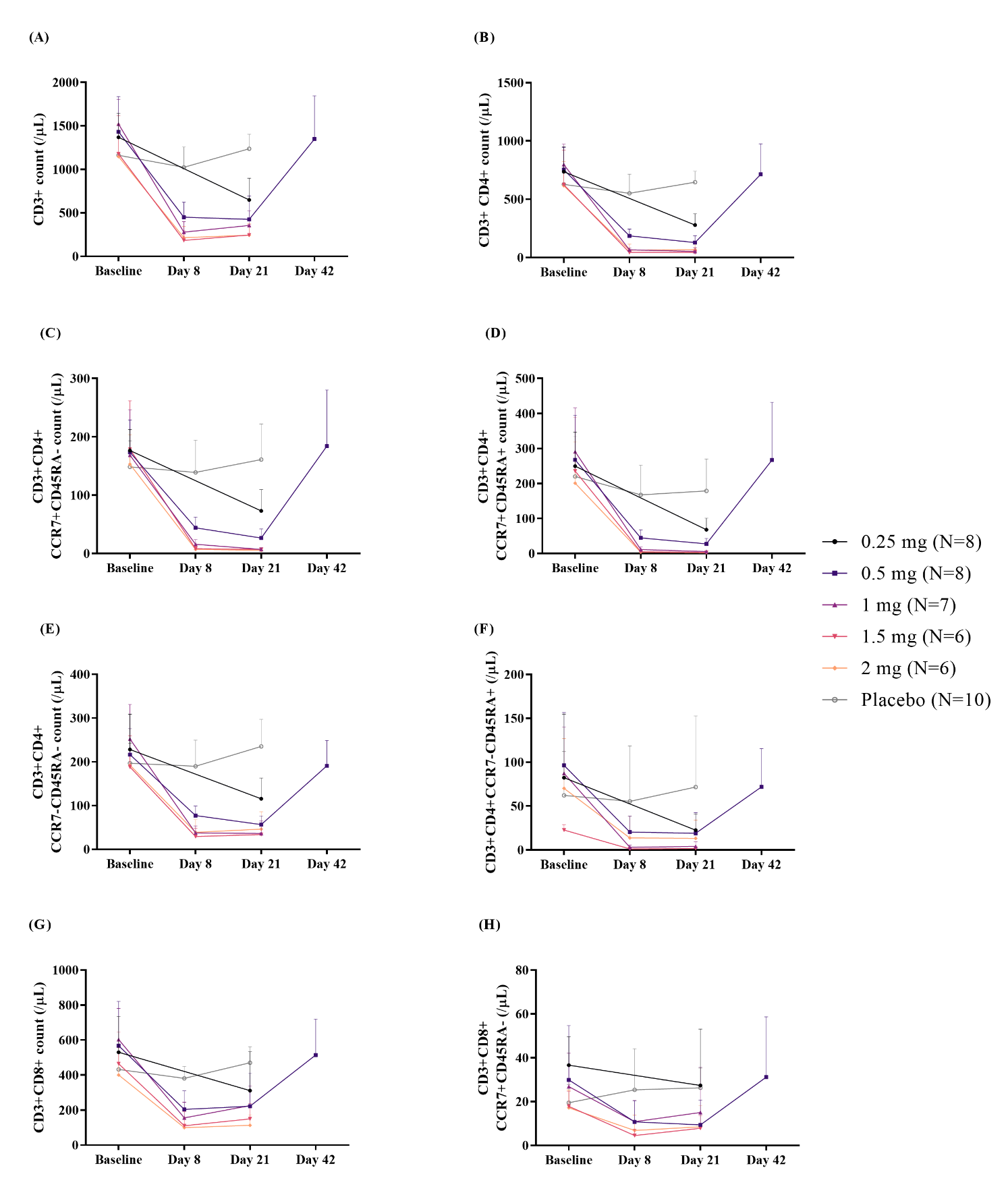

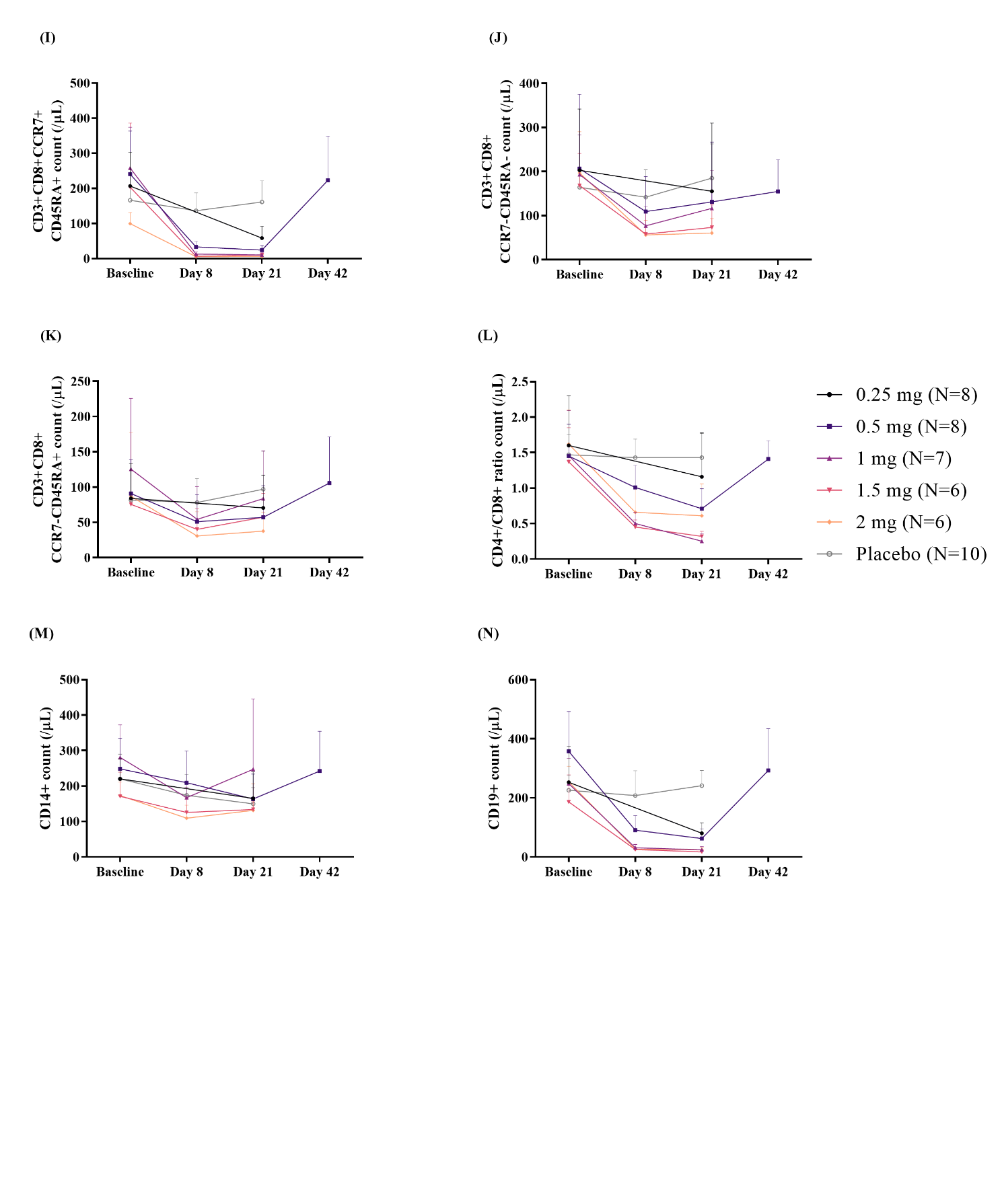


**Supplementary Figure 5.** Mean (A) CD3+ (B) CD3+CD4+ (C) CD3+ CD4+ CCR7+ CD45RA- (D) CD3+ CD4+ CCR7+ CD45RA+ (E) CD3+ CD4+ CCR7- CD45RA- (F) CD3+ CD4+ CCR7+ CD45RA- (G) CD3+ CD8+ (H) CD3+ CD8+ CCR7+ CD45RA- (I) CD3+ CD8+ CCR7+ CD45RA+ (J) CD3+ CD8+ CCR7- CD45RA- (K) CD3+ CD8+ CCR7+ CD45RA- (L) CD4+/CD8+ ratio (M) CD14+ (N) CD19+ count after single and daily oral administration of 0.25 mg, 0.5 mg, 1 mg, 1.5 mg, and 2 mg LC51-0255 for 21 days.

Supplementary Table 1. Receptor specificity of LC51-0255 compared to other S1P receptor modulators via *in vitro* Ca^2+^ mobilization assay

| **S1P receptor subtype** | **EC50 (nM)** | | |
| --- | --- | --- | --- |
|  | **Fingolimod** | **Siponimod** | **LC51-0255** |
| **S1PR1** | 6.61 | 39.81 | 21.38 |
| **S1PR3** | 24.5 | >10000 | >10000 |
| **S1PR5** | >10000 | 741.31 | 501.19 |

**Notes:** EC50 is defined as the concentration of each compound corresponding to 50% of the maximum activity (E_max_) when treated with 1 uM of S1P.
**Abbreviations:** S1P, sphingosine-1-phosphate.

Supplementary table 2 Details of antibodies used in fluorescence-activated cell sorting (FACS) analysis.

| **Group** | **Product name** | **Antibody** | **Fluorescence** | **Catalog number** |
| --- | --- | --- | --- | --- |
| **Group I** | PerCP-Cy5.5 Mouse Anti-Human CD3 | CD3 | PerCP-CyTM5.5 | 332771 |
|  | PE Mouse Anti-Human CD4 | CD4 | PE | 345769 |
|  | APC Mouse Anti-Human CD8 | CD8 | APC | 345775 |
|  | FITC Mouse Anti-Human CD45RA | CD45RA | FITC | 335039 |
|  | PE-Cy7 Rat Anti-Human CD197 (CCR7) | CD197(CCR7) | PE-CyTM7 | 557648 |
|  | CD45 APC-H7 | CD45 | APC-H7 | 641417 |
| **Group II** | CD14 APC-Cy7 CE | CD14 | APC-CyTM7 | 333951 |
|  | CD19 PE-Cy7 CE | CD19 | PE-CyTM7 | 341113 |
|  | APC Mouse Anti-Human CD138 | CD138 | APC | 347216 |
|  | Tritest CD3/CD16+CD56/CD45 W/Trucount Tubes | CD3 | FITC | 340403 |
|  |  | CD16+56+ | PE |  |
|  |  | CD45 | PerCP |  |
| **QC** | 6-Color TBNK Reagent with BD Trucount™ | CD3 | FITC | 337166 |
|  |  | CD4 | PE-CyTM7 |  |
|  |  | CD8 | APC-CyTM7 |  |
|  |  | CD45 | PerCP-CyTM5.5 |  |
|  |  | CD16+56+ | PE |  |
|  | BD Multi-Check control | - | - | 340911 |
|  | BD Multi-Check CD4 Low Ctrl | - | - | 340914 |

Supplementary table 3 Summary of treatment-emergent adverse events after daily administrations of 0.25 mg, 0.5 mg, 1 mg, 1.5 mg, 2 mg LC51-0255 and placebo for 21 days.

| **Treatment-emergent adverse events** | **Placebo (N = 10)** | **LC51-0255** | | | | | |
| --- | --- | --- | --- | --- | --- | --- | --- |
|  |  | **0.25 mg (N = 8)** | **0.5 mg (N = 8)** | **1 mg (N = 8)** | **1.5 mg (N = 8)** | **2 mg (N = 8)** | **Total (N = 40)** |
| **Number of subjects with TEAEs** | 5 (50%) [15] | 6 (75%) [9] | 5 (62.5%) [12] | 6 (75%) [13] | 6 (75%) [23] | 6 (75%) [17] | 29 (72.5%) [74] |
| **Bradycardia** | 0 (0%) [0] | 1 (12.5%) [1] | 1 (12.5%) [1] | 1 (12.5%) [3] | 2 (25%) [4] | 3 (37.5%) [5] | 8 (20%) [14] |
| **Dizziness** | 1 (10%) [1] | 2 (25%) [2] | 0 (0%) [0] | 3 (37.5%) [3] | 2 (25%) [2] | 1 (12.5%) [1] | 8 (20%) [8] |
| **Headache** | 2 (20%) [2] | 0 (0%) [0] | 2 (25%) [2] | 0 (0%) [0] | 0 (0%) [0] | 3 (37.5%) [4] | 5 (12.5%) [6] |
| **Aphthous ulcer** | 0 (0%) [0] | 0 (0%) [0] | 1 (12.5%) [1] | 2 (25%) [2] | 0 (0%) [0] | 0 (0%) [0] | 3 (7.5%) [3] |
| **Arthralgia** | 0 (0%) [0] | 1 (12.5%) [1] | 1 (12.5%) [1] | 0 (0%) [0] | 1 (12.5%) [1] | 0 (0%) [0] | 3 (7.5%) [3] |
| **Diarrhoea** | 1 (10%) [1] | 0 (0%) [0] | 1 (12.5%) [1] | 1 (12.5%) [1] | 0 (0%) [0] | 1 (12.5%) [1] | 3 (7.5%) [3] |
| **Oropharyngeal pain** | 1 (10%) [1] | 1 (12.5%) [1] | 1 (12.5%) [1] | 0 (0%) [0] | 1 (12.5%) [1] | 0 (0%) [0] | 3 (7.5%) [3] |
| **Rhinorrhoea** | 1 (10%) [1] | 0 (0%) [0] | 0 (0%) [0] | 1 (12.5%) [1] | 2 (25%) [2] | 0 (0%) [0] | 3 (7.5%) [3] |
| **Abdominal pain** | 1 (10%) [1] | 0 (0%) [0] | 1 (12.5%) [1] | 0 (0%) [0] | 0 (0%) [0] | 1 (12.5%) [1] | 2 (5%) [2] |
| **Chest discomfort** | 0 (0%) [0] | 0 (0%) [0] | 0 (0%) [0] | 0 (0%) [0] | 1 (12.5%) [1] | 1 (12.5%) [1] | 2 (5%) [2] |
| **Fatigue** | 0 (0%) [0] | 0 (0%) [0] | 0 (0%) [0] | 0 (0%) [0] | 1 (12.5%) [1] | 1 (12.5%) [1] | 2 (5%) [2] |
| **Toothache** | 0 (0%) [0] | 0 (0%) [0] | 0 (0%) [0] | 0 (0%) [0] | 2 (25%) [2] | 0 (0%) [0] | 2 (5%) [2] |

Notes: Treatment-emergent adverse events with two or more cases are shown in the table. Denominator of percentage is the number of subjects in the column. Data regarding number of subjects with TEAEs are presented as number of subjects (percentage of subjects) [number of events].
Abbreviations: TEAE, treatment-emergent adverse event.
